# Persistence and remission of depressive and generalized anxiety symptoms among adolescent mothers in Mwanza, Tanzania: a longitudinal observational study embedded within a non-pharmacological intervention cohort

**DOI:** 10.3389/fgwh.2026.1814117

**Published:** 2026-10-28

**Authors:** Matiko Mwita, Deborah Dewey, Eveline T. Konje, Scott Patten

**Affiliations:** 1 Psychiatry Department, Catholic University of Health and Allied Sciences (CUHAS), Mwanza, Tanzania; 2 Psychiatry Department, Bugando Medical Centre (BMC), Mwanza, Tanzania; 3 Departments of Pediatrics and Community Health Sciences, Owerko Centre at the Alberta Children’s Hospital Research Institute, Hotchkiss Brain Institute, University of Calgary, Calgary, CA, Canada; 4 Department of Epidemiology and Biostatistics, Catholic University of Health and Allied Sciences (CUHAS), Mwanza, Tanzania; 5 Cuthbertson & Fisher Chair, Departments of Community Health Sciences and Psychiatry, University of Calgary, Calgary, CA, Canada

**Keywords:** adolescent pregnancy, anxiety, depression, mental health, perinatal, remission

## Abstract

**Background:**

There is paucity of literature on the persistence and remission rates of depressive and anxiety symptoms during pregnancy and to our knowledge, no studies have examined these trajectories among adolescent mothers. Moreover, associated psychosocial risk and protective factors remain largely underexplored. To begin to address these gaps, we used longitudinal data from a cohort of pregnant adolescents in Mwanza, Tanzania, who participated in a non-pharmacological intervention to examine the remission and persistence of depressive and generalized anxiety symptoms from pregnancy through to three months postpartum.

**Methods:**

A total of 533 pregnant adolescents were enrolled during their second trimester using a convenience sampling approach from selected antenatal care clinics in Mwanza, Tanzania, between 5 August 2023 and 30 June 2024. Depressive symptoms were assessed using the Edinburgh Postnatal Depression Scale (EPDS) and generalized anxiety symptoms were assessed using the Generalized Anxiety Disorder–7 (GAD-7) scale. Assessments were conducted at four time points: second trimester (Time 1), third trimester (Time 2), four weeks postpartum (Time 3), and three months postpartum (Time 4). Participants who screened positive for depressive and/or anxiety symptoms at Time 1, the initial assessment were followed longitudinally to three months post-delivery.

**Results:**

Approximately one-third of adolescent mothers who screened positive for depressive or anxiety symptoms at Time 1, continued to report symptoms in the third trimester. Symptom remission rates were high postpartum, with more than 97% of participants who screened positive for depressive and anxiety symptoms at Time 1 reporting remission at three months post-delivery.

**Conclusions:**

Maternal depressive and anxiety symptoms in adolescent mothers were dynamic, showing moderate persistence during pregnancy and marked remission in the postpartum period. These findings support the importance of integrating routine mental health screening alongside sustained psychosocial interventions into antenatal and postnatal care to reduce the burden of perinatal mental disorders among adolescent mothers in low-resource settings.

## Introduction

Depression and anxiety are among the most common mental health disorders affecting adolescents globally, with a disproportionate burden in low- and middle-income countries (LMICs) (1). Adolescent pregnancy may further increase vulnerability to poor mental health, as young mothers experience intersecting biological, psychological, and social stressors during a critical developmental period  (2). Depression and anxiety during pregnancy and the postpartum period can adversely affect maternal functioning and mother–infant bonding, and are associated with poorer infant and long-term maternal outcomes (3).

Tanzania has one of the highest rates of adolescent pregnancy globally, with approximately 27% of adolescent women aged 15–19 years having initiated childbearing (4, 5). In sub-Saharan Africa (SSA), adolescent pregnancy often occurs in the contexts of socioeconomic disadvantage and stigma, which may compound psychosocial stress and vulnerability to poor mental health (6). In Mwanza, Tanzania, structural challenges including poverty, limited access to reproductive health services, and school dropout may further contribute to the psychosocial stress and the mental health challenges experienced by adolescent mothers (7). Despite the substantial burden of adolescent pregnancy in SSA, longitudinal evidence on the trajectories of persistence and remission of depressive and anxiety symptoms among adolescent mothers remains limited.

Evidence from high income countries indicates that perinatal depressive and anxiety symptoms can persist or remit over time and their course may be associated with individual or psychosocial characteristics. For example, among pregnant women in the Netherlands with a history of depression and had used antidepressants, but no current diagnosis of depression, Molenaar et al. reported that 9.4% experienced a recurrence of depression, two during pregnancy and six in the first 12 weeks postpartum (8). In a study of Chinese immigrant and Canadian-born women with depressive and/or anxiety symptomatology at 4 weeks postpartum, Dennis et al. found that a substantial proportion experienced persistence of symptoms. Specifically, 40.0% of women with possible depressive symptoms continued to have symptoms at 12 weeks postpartum and 15.8% at 52 weeks postpartum. Among women with high depressive symptomatology, 28.2% continued experience symptoms at 12 weeks and 6.1% at 52 weeks. Persistence of anxiety symptoms was observed in 42.0% of women at 12 weeks and 17.4% at 52 weeks postpartum, while persistence of comorbid depression and anxiety symptoms was observed in 30.0% and 8.9% of women at 12 at 52 weeks postpartum, respectively (9). These studies also suggest that psychosocial characteristics including age, parity, educational level, occupation and pregnancy planning were associated with the course of depressive and anxiety symptoms (8, 9).

Understanding the persistence and remission of depressive and anxiety symptoms among adolescent mothers is particularly important in settings where adolescent pregnancy is common and access to mental health services may be limited. Identifying psychosocial factors associated with different symptom trajectories may help inform the timing and targeting of mental health screening and support. However, longitudinal studies examining the course of depressive and anxiety symptoms among adolescent mothers in SSA remain scarce and evidence from Tanzania is particularly limited. To our knowledge, no previous studies have systematically examined patterns of persistence and remission of depressive and anxiety symptoms among pregnant adolescents across pregnancy and the postpartum period in Tanzania.

To address this gap, we analyzed longitudinal data from a cohort of pregnant adolescents in Mwanza, Tanzania (10). Depressive and generalized anxiety symptoms were assessed at multiple time points from the second trimester of pregnancy through three months postpartum. We aimed to: (1) characterize patterns persistence and remission of depressive and anxiety symptoms across the perinatal period and (2) identify psychosocial factors associated with these symptom trajectories. By examining the course of symptoms longitudinally, this study provides evidence on perinatal mental health among adolescent mothers in an understudied SSA population and may help inform approaches to identifying adolescents who could benefit from targeted mental health support.

## Materials and methods

### Study design and settings

The study design and setting has been described previously (10). Briefly, pregnant adolescents were enrolled during their second trimester, which is typically when most pregnant women in Tanzania make their initial antenatal clinic (ANC) visit. Recruitment was conducted at three purposively selected ANCs in the Mwanza region: Makongoro and Butimba clinics in Nyamagana district, and Buzuruga clinic in Ilemela district. These facilities are among the largest government-run health centers in the Mwanza and offer comprehensive antenatal, postnatal, and reproductive and child health (RCH) services. Each clinic provides care to approximately 500 pregnant and postpartum women per month, including approximately 30 adolescents (11). Participant characteristics did not vary meaningfully across the three recruitment sites.

### Participants and recruitment methods

A convenience sampling approach was used. All adolescent pregnant woman who presented consecutively at the reception desks of the three participating ANC clinics were invited to participate. An *a priori* power analysis estimated that 476 participants would provide 80% power to detect a prespecified effect in depressive symptoms over time, at a two-sided 5% significance level, assuming a baseline depression prevalence of 32.9% (12). To account for potential loss to follow-up, the target sample was increased by at least 10%, resulting in a planned enrollment of 533 participants. Recruitment and follow-up occurred between 5 August 2023 and 30 June 2024.

Eligible adolescents who agreed to participate provided written informed consent. For participants younger than 18 years, written assent was obtained from the adolescent, and written consent was obtained from the parent or legal guardian, including a spouse where applicable. Adolescents who were visibly unwell at the time of recruitment period were not approached. Overall, the study achieved a participation rate of approximately 99%, with nearly all eligible adolescent invited to participate providing consent.

### Procedure

Trained research assistants approached pregnant adolescents attending the participating antenatal care (ANC) clinics and invited them to participate in the study. Following informed consent or assent and parent/legal guardian consent, as applicable, participants completed interviewer-administered assessments. Depressive and anxiety symptoms were assessed at four scheduled time points: during the second trimester at enrollment (T1), during the third trimester (T2), at approximately four weeks postpartum (T3), and at three months postpartum (T4). The T1 assessment served as the baseline assessment for all participants. All assessments were conducted in the ANC clinic setting. The same research assistant conducted assessments for an individual participant across study visits to promote consistency in administration.

Participants who screened positive for depressive and/or anxiety symptoms at T1, T2, or T3 were invited to participate in a non-pharmacological psychosocial intervention. Participants could therefore enter the intervention at enrollment (T1) or following symptom screening at T2 or T3. Group sessions were facilitated by trained nurse-midwives and integrated with routine antenatal and postnatal care appointments and were offered approximately once per month.

### Non-pharmacological psychosocial intervention

The non-pharmacological psychosocial intervention was based on the World Health Organization (WHO) Thinking Healthy Programme (THP) and adapted for adolescents in Tanzania. The adaptions added locally relevant content, components of cognitive behavior therapy (CBT) and interpersonal therapy (IPT), and a focus on both depressive and anxiety symptoms, and was refined to ensure its appropriateness and feasibility for adolescents’ management of perinatal depression (13–15).

The adolescent who participated in the intervention first received an individual psychotherapy session delivered by the principal investigator, a clinical psychiatrist that focused on emotional regulation and coping skills. They then joined group psychotherapy sessions consisting of approximately 6–8 adolescent mothers. Groups were organized according to geographical location and other practical considerations, including the timing of participant identification. Sessions were facilitated by trained nurse-midwives, integrated with routine antenatal and postnatal appointments, and held approximately monthly and participating adolescents attended a minimum of eight sessions.

Of the 533 adolescents enrolled, 110 screened positive for depressive symptoms and 119 screened positive for anxiety symptoms at T1 and were longitudinally followed to examine symptom remission or persistence through to T3. Adolescents who were not symptomatic at T1, but who screened positive at T2 or T3 were invited to participate in the intervention. Overall, 317 adolescents screened positive for depressive and/or anxiety symptoms at one or more assessments (T1–T3) and participated in the intervention.

### Measures

Participants completed a sociodemographic questionnaire that captured details such as age, education, marital status, household composition, employment, pregnancy planning, and history of partner violence. Depression and anxiety symptoms were assessed using the Edinburgh Postnatal Depression Scale (EPDS) and the Generalized Anxiety Disorder–7 (GAD-7), respectively Although the questionnaires were designed to be self-administered, trained research assistants read each item aloud in Swahili, that national language of Tanzania, and recorded participants’ responses to accommodate participants’ needs and ensure standardized administration.

The EPDS is a 10-item measure assessing depressive symptoms experienced during the previous week. Each item is scored from 0 to 3, yielding a total score ranging from 0 to 30. Scores of 0–8 generally indicate low likelihood of depression, 9–11 possible depression, 12–13 a high likelihood of depression, and 14 or above probable depression. In the present study, an EPDS score of ≥ 9 was used to indicate depressive symptoms (16). Although a cut off of ≥9 has been supported for identifying identify perinatal depression symptoms in some high income settings (17), research conducted in LMIC countries reported that a slightly higher threshold of ≥10 may provide better classification (18). A cut-off of ≥9 was selected in the present study to maximize sensitivity for identifying adolescents who may benefit from psychosocial support.

The GAD-7 is a seven-item measure assessing the frequency of anxiety symptoms during the previous two weeks. Items are rated on a four-point scale scored from 0 to 3, yielding a total score ranging from 0 to 21. Scores of 0–4 indicate minimal anxiety, 5–9 mild anxiety, 10–14 moderate anxiety, and 15–21 severe anxiety. In this study, a GAD-7 ≥ 5 was used to indicate symptoms of generalized anxiety (19) consistent with previous research indicating this threshold for identifying increased likelihood of generalized anxiety disorders in perinatal populations (20).

Both the EPDS and GAD-7 demonstrate strong internal reliability, with Cronbach’s alpha values of 0.83 (21) and 0.82 (22), respectively.

### Data management and analysis

Descriptive statistics were used to summarize participant characteristics and symptom measures. Frequencies and percentages were calculated for categorical variables, while means and standard deviations were computed for continuous measures.

Persistence and remission of depressive and generalized anxiety symptoms were examined longitudinally across the four assessment time points. Persistence of depressive or anxiety symptoms was defined as continuing to meet the prespecified threshold at a subsequent follow-up assessment (T2, T3, or T4) among participants who met the threshold at baseline (T1). Remission was defined as no longer meeting the prespecified threshold for depressive or anxiety symptoms at a subsequent assessment among participants who met the threshold at baseline. The EPDS cut-off of ≥9 was used to identify depressive symptoms, and the GAD-7 cut-off of ≥5 was used to identify anxiety symptoms.

Univariate logistic regressions were conducted to examine associations between sociodemographic and psychosocial factors and depressive and anxiety symptoms at different time points. Candidate predictors included age (19 years versus younger), education level (secondary level or higher, versus lower educational level), marital status (married versus single), occupation (employed versus not employed), living situation (living with their partner or living with parents/caretakers). whether the pregnancy was planned (planned versus unplanned), and history of partner violence (experienced violence or did not experience violence). Outcomes were analyzed as binary variables indicating the presence or absence of depressive or anxiety symptoms. Odds ratios (ORs) and corresponding 95% confidence intervals (CIs) were reported. All statistical analyses were performed using Stata version 15 for Mac.

### Ethical approval

The study protocol and publication of its findings were approved by the Catholic University of Health and Allied Sciences/Bugando Medical Centre Joint Ethical Committee (CREC/668/2023) and the University of Calgary (REB23-0674). The study was conducted in accordance with the ethical principles of the 1964 Declaration of Helsinki. Permission to conduct this study was obtained from the regional administrative and local government authorities.

Participant confidentiality was maintained throughout data collection and analysis. Participants were identified using study identification numbers rather than names on study questionnaires and analytical datasets.

## Results

### Sociodemographic and pregnancy related characteristics

At enrollment, participants had an average age of 18.29 years (SD = 0.90; range 15–19 years), with roughly half being 19 years old (52.72%, *n* = 281). Almost 2/3 were married (65.67%, *n* = 350), with 2/3 of the participants reporting to be living with their partners (66.23%, *n* = 353) regardless if legally married or not. Most were experiencing their first pregnancy (primigravida) (88.56%, *n* = 472), and about half reported that the pregnancy had been planned (50.09%, *n* = 267). Sociodemographic and pregnancy related characteristics of the sample are presented in Table 1.

**Table 1:** Sociodemographic and pregnancy related characteristics of the study participants (*N* = 533).

Variable	Frequency (*n*)	Percentage (%)
Age of pregnant adolescents (years)		
<18	91	17.07
18	161	30.21
19	281	52.72
Marital status		
Not married	183	34.33
Married	350	65.67
Who do you live with?		
With partner	353	66.23
With parents/caretakers	180	33.77
Education level		
Never went to school	35	6.57
Primary education	286	53.66
Secondary education	212	39.77
Employment status		
Never employed	444	83.30
Employed	89	16.70
Planned pregnancy		
No	266	49.91
Yes	267	50.09
Do you encounter any form of partner violence?		
No	497	93.25
Yes	36	6.75

### Persistence and remission rates of depressive and anxiety symptoms

Among women with depressive symptoms at the second trimester of pregnancy (T1), 30.0% (95% CI, 23.2–32.2) continued to report symptoms at the third trimester (T2). This proportion declined substantially postpartum, with 3.6% (95% CI, 2.9–5.5) reporting persistent symptoms at four weeks post-delivery (T3) and 2.8% (95% CI, 1.8–3.2) at three months post-delivery (T4). Similarly, among those with anxiety symptoms at T1, 30.3% (95% CI, 24.8–33.1) had persistent symptoms at T2, decreasing to 4.2% (95% CI, 3.7–6.6) at T3 and 3.4% (95% CI, 2.6–4.1) at T4. Remission was high for both depressive and anxiety symptoms, with approximately 70% of participants who screened positive at the second trimester remitting by the third trimester and more than 97% remitting at four weeks and three months post-delivery. Persistent and remission rates of depressive and anxiety symptoms at different perinatal time points are presented in Table 2.

**Table 2:** Persistent and remission rates of depressive and anxiety symptoms at different perinatal time points.

	Time 1: 2nd trimester Pregnancy	Time 2: 3rd trimester pregnancy		Time 3: 4 weeks post delivery		Time 4: 3 months post delivery	
		Persistence	Remission	Persistence	Remission	Persistence	Remission
Depressive symptoms	*N* = 110	33/110 30.0% (23.2–32.2)	77/110 70.0% (67.3–73.1)	4/110 3.6% (2.9–5.5)	106/110 96.4% (94.9–98.4)	3/108 2.8% (1.8–3.2)	105/108 97.2% (96.6–98.2)
Anxiety symptoms	*N* = 119	36/119 30.3% (24.8–33.1)	83/119 69.7% (68.2–71.5)	5/119 4.2% (3.7–6.6)	114/119 95.8% (93.2–97.9)	4/116 3.4% (2.6–4.1)	112/116 96.6% (95.4–98.5)

Variation in denominators across time points reflects missing outcome data for individual participants for reasons unrelated to loss to follow-up.

### Associations between remission of depressive symptoms and sociodemographic factors across time points

Given the small number of participants who remained symptomatic at subsequent assessments, multivariable models were not considered sufficiently stable; therefore, univariable logistic regression models were used to examine associations between participant characteristics and remission. The results represent unadjusted associations and should not be interpreted as independent predictors of remission.

From the third trimester (Time 2), being 19 years of age compared to being younger, was consistently associated with higher odds of remission of depressive symptoms with the strength of association increasing over time (Time 2: OR=2.2, 95% CI: 1.9–3.7, *p* = 0.001; Time 3: OR=3.2, 95% CI: 2.1–3.9, *p* = 0.021; Time 4: OR=3.5, 95% CI: 2.9–3.9, *p* = 0.012). Similarly, being married was associated with greater likelihood of remission across each follow-up time points (ORs ranging from 1.2 at Time 2 to 1.8, at Time 4, all *p* < 0.05). A planned pregnancy was also a significant predictor of remission during the third trimester (OR=1.5, 95% CI: 1.4–1.8, *p* = 0.039), at 4 weeks postpartum (OR=1.7, 95% CI: 1.4–2.9, *p* = 0.001), and at 3 months postpartum (OR=1.8, 95% CI: 1.5–2.2, *p* = 0.024).

Conversely, living with parents or caretakers was associated with reduced odds of remission at each follow-up points (ORs ranging from 0.4 at Time 2 to 0.6 at Time 4, each *p* < 0.05). Experiencing partner violence was also consistently associated with lower odds of remission during pregnancy and the postpartum period, with particularly strong effects observed at 4 weeks postpartum (Time 3) (OR=0.2, 95% CI: 0.1–0.4, *p* < 0.001). Educational attainment and employment status were not significantly associated with remission of depressive symptoms at any time point. Associations between remission of depressive symptoms and sociodemographic factors across time points are presented in Table 3.

**Table 3:** Associations between remission of depressive symptoms and sociodemographic variables at different time points.

Variable	Association with depressive symptoms at Time 1: 2nd trimester Pregnancy		Univariable associations between selected factors and remission of depressive symptoms					
			Time 2: 3rd trimester Pregnancy		Time 3: 4 weeks post delivery		Time 4: 3 months post delivery	
	OR (95% CI)	*P* value	OR (95% CI)	*P* value	OR (95% CI)	*P* value	OR (95% CI)	*P* value
19 years of age	0.4 (0.2–0.7)	0.001	2.2 (1.9–3.7)	0.001	3.2 (2.1–3.9)	0.021	3.5 (2.9–3.9)	0.012
Being Married	0.3 (0.2–0.5)	<0.001	1.2 (1.1–1.4)	0.030	1.7 (1.5–2.1)	0.008	1.8 (1.3–1.9)	0.009
Living With parents/caretakers	3.8 (2.4–5.8)	<0.001	0.6 (0.5–0.9)	0.042	0.6 (0.2–0.9)	0.031	0.4 (0.2–0.6)	0.043
≥ Secondary education	0.9 (0.6–1.2)	0.526	0.4 (0.2–1.8)	0.542	0.8 (0.5–1.6)	0.631	1.1 (0.9–1.9)	0.285
Employed	0.3 (0.1–1.6)	0.510	1.0 (0.6–2.7)	0.486	0.4 (0.3–1.8)	0.152	0.3 (0.2–2.6)	0.631
Planned pregnancy	0.2 (0.1–0.4)	<0.001	1.5 (1.4–1.8)	0.039	1.7 (1.4–2.9)	0.001	1.8 (1.5–2.2)	0.024
Experiencing any form of partner violence	3.4 (1.7–6.7)	0.001	0.8 (0.4–0.9)	0.002	0.2 (0.1–0.4)	<0.001	0.6 (0.4–0.8)	0.001

Associations between persistence of depressive symptoms and sociodemographic factors across time points are not reported because the small number of adolescent mothers who reported depressive symptoms postpartum resulted in unstable and unreliable estimates.

### Associations between remission of anxiety symptoms and sociodemographic factors across time points

Given the small number of participants who remained symptomatic at subsequent assessments, multivariable models were not considered sufficiently stable; therefore, univariable logistic regression models were used to examine associations between participant characteristics and remission. The results represent unadjusted associations and should not be interpreted as independent predictors of remission.

From the third trimester (Time 2) onward, being 19 years of age was consistently associated with increased odds of remission of anxiety symptoms (compared to being younger), with the strength of association increasing over time (Time 2: OR=1.3, 95% CI: 1.2–2.1, *p* = 0.002; Time 3: OR=2.1, 95% CI: 1.9–2.6, *p* = 0.013; Time 4: OR=2.5, 95% CI: 2.2–2.9, *p* = 0.007). Similarly, being married was associated with higher odds of remission at each follow-up time points (ORs ranging from 1.2 at Time 2 to 1.5 at Time 4, all *p* < 0.05). Planned pregnancy was also a significant predictor of remission during the third trimester (OR=1.3, 95% CI: 1.1–1.6, *p* = 0.020), at 4 weeks postpartum (OR=1.5, 95% CI: 1.4–2.0, *p* = 0.005), and at 3 months postpartum (OR=1.6, 95% CI: 1.3–2.1, *p* = 0.016).

Conversely, living with parents or caretakers was consistently associated with reduced odds of anxiety symptom remission across each three follow-up periods (ORs ranging from 0.3 at Time 2 to 0.6 at Time 4, each *p* < 0.05). Experiencing partner violence was also associated with lower likelihood of remission during pregnancy and the postpartum period (ORs ranging from 0.6 at Time 2 to 0.7 at Time 4, each *p* ≤ 0.002). Educational attainment at secondary level or higher and employment status were not significantly associated with remission of anxiety symptoms at any time point. Associations between remission of anxiety symptoms and sociodemographic factors across time points are presented in Table 4.

**Table 4:** Associations between remission of anxiety symptoms and sociodemographic variables at different time points.

Variable	Association with anxiety symptoms at Time 1: 2nd trimester Pregnancy		Univariable associations between selected factors and remission of anxiety symptoms					
			Time 2: 3rd trimester Pregnancy		Time 3: 4 weeks post delivery		Time 4: 3 months post delivery	
	OR (95% CI)	*P* value	OR (95% CI)	*P* value	OR (95% CI)	*P* value	OR (95% CI)	*P* value
19 years of age	0.7 (0.4–1.2)	0.168	1.3 (1.2–2.1)	0.002	2.1 (1.9–2.6)	0.013	2.5 (2.2–2.9)	0.007
Being Married	0.7 (0.1–0.9)	0.043	1.2 (1.1–1.4)	0.032	1.5 (1.2–1.8)	0.017	1.2 (1.1–1.5)	0.028
Living With parents/caretakers	10.3 (7.5–12.3)	0.016	0.3 (0.1–0.6)	0.029	0.4 (0.2–0.8)	0.011	0.6 (0.3–0.9)	0.031
≥ Secondary education	0.8 (0.4–2.0)	0.696	0.5 (0.2–1.6)	0.492	0.6 (0.4–1.3)	0.361	1.4 (0.8–1.7)	0.385
Employed	0.9 (0.5–1.6)	0.776	1.3 (0.8–2.1)	0.816	1.2 (0.1–1.7)	0.241	0.4 (0.1–4.6)	0.920
Planned pregnancy	0.4 (0.3–0.8)	0.005	1.3 (1.1–1.6)	0.020	1.5 (1.4–2.0)	0.005	1.6 (1.3–2.1)	0.016
Experiencing any form of partner violence	4.6 (2.1–9.9)	<0.001	0.6 (0.2–0.9)	0.002	0.7 (0.4–0.9)	<0.001	0.7 (0.4–0.8)	0.001

Again, associations between persistence of anxiety symptoms and sociodemographic factors across time points are not reported because the small number of adolescent mothers who reported anxiety symptoms postpartum resulted in unstable and unreliable estimates.

## Discussion

This longitudinal study of adolescent mothers attending antenatal care (ANC) clinics in Mwanza, Tanzania, examined the persistence and remission of depressive and generalized anxiety symptoms from pregnancy through the early postpartum period, as well as psychosocial risk and protective factors associated with symptom remission. Overall, the findings indicate a dynamic pattern of perinatal mental health, characterized by moderate persistence of symptoms during pregnancy and a marked reduction in symptoms following delivery. The provision of a psychological intervention to all participants who screened positive may have contributed to the low prevalence of persistent symptoms in the postpartum period, although the study design does not permit causal inference regarding its effects. Younger age, marital status, pregnancy intentionality, living arrangements, and exposure to partner violence were also associated with symptom remission trajectories, highlighting the potential importance of both individual and social-contextual factors in shaping mental health trajectories among adolescent mothers.

We observed that approximately one-third of adolescent mothers who reported depressive or anxiety symptoms during the second trimester continued to experience symptoms in the third trimester, indicating substantial symptom persistence during pregnancy. In contrast, symptom persistence declined markedly in the postpartum period, with fewer than 5% of participants reporting ongoing depressive or anxiety symptoms at four weeks and three months after delivery. This pattern differs notably from findings in longitudinal studies of adult perinatal populations, in which depressive and anxiety symptoms often persist or intensify following childbirth (23–25). Studies from high-income settings have reported that up to 40% of women with early postpartum depressive symptoms continue to report symptoms at three months postpartum, and a substantial proportion remaining symptomatic up to one year after delivery (9).

The comparatively low persistence of depressive and anxiety symptoms in the post-partum period may reflect a combination of factors including physiological and psychosocial changes following childbirth, relief from pregnancy-related stressors, and the structured psychological intervention offered to adolescents who screened positive. Evidence from randomized and observational studies suggests that early and sustained psychological support during pregnancy and postpartum can reduce symptom severity, facilitate remission, and potentially prevent chronicity of perinatal depression and anxiety (26, 27). In low-resource settings, task-shifted and clinic-based psychological interventions have been shown to be feasible and effective, particularly when embedded within routine maternal care (28). These findings provide a plausible context for the low levels of persistent symptoms observed in the present cohort, although the absence of an untreated comparison group precludes determining the extent to which the intervention contributed to the observed postpartum decline.

Regression to the mean may also have contributed to the observed reduction in depressive and anxiety symptoms over follow-up. Because participants were identified based on elevated symptom-screening scores, some subsequent reduction may reflect the natural variability of symptoms and measurement rather than substantive change in symptom burden. Thus, the observed remission may reflect a combination of postpartum physiological and psychosocial changes, intervention exposure, and regression to the mean. However, in the absence of an untreated comparison group, the relative contribution of these factors cannot be determined.

In this cohort, all adolescents who screened positive for depressive or anxiety symptoms at their initial assessment were invited to participate in the psychological intervention that extended through to three months postpartum. The integration of psychological support across pregnancy and the postpartum period may have contributed to the observed decline in symptom persistence after delivery. Previous studies have shown that psychological interventions—including counseling, psychoeducation, and peer support—can be of benefit for adolescents and first-time mothers, who may have limited coping resources and heightened vulnerability to perinatal mental health difficulties (26, 29). Sustained support in the postpartum period may be particularly important, as this is a critical window for preventing persistent and recurring symptoms and promoting maternal–infant well-being (30). However, because all eligible participants were offered the intervention and there was no untreated comparison group, its specific contribution to the observed symptom trajectories cannot be determined.

Nonetheless, a small subgroup of participants continued to experience symptoms postpartum, underscoring the need for tailored care pathways, including effective referral mechanisms to general, and when indicated specialized mental health care services. Adolescents experiencing persistent symptoms or ongoing psychosocial stressors may require more intensive or longer-term mental health support beyond the early postpartum period to reduce depression and anxiety symptoms. Strengthening continuity of care and referral pathways may therefore be important for ensuring that those with persistent or worsening symptoms receive appropriate assessment and treatment.

Several psychosocial factors were consistently associated with remission of both depressive and anxiety symptoms. From the third trimester onward, being 19 years of age was associated with significantly higher odds of remission compared with being younger, with the strength of this association increasing over time. Older adolescents may have greater emotional maturity, autonomy, and problem-solving capacity, which may facilitate adaptation to pregnancy and motherhood, and in turn contribute to symptom resolution (31, 32). These findings are consistent with previous research suggesting that younger adolescents be at greater risk of persistent perinatal mental health problems because of developmental, social, and economic vulnerabilities (33).

Being married was also associated with greater likelihood of symptom remission across each time point. Marriage may provide emotional, financial, and practical support, which may buffer stress and facilitate recovery from perinatal mood and anxiety disorders (34, 35). In contrast, living with parents or other was associated with associated with reduced odds of remission for both depressive and anxiety symptoms. Although extended family living arrangements can provide important social and practical support, they may also be associated with reduced autonomy, intergenerational conflict, or social control, particularly for adolescent mothers (36). Such circumstances may contribute to ongoing psychosocial stress, and potentially limit recovery, even when formal psychosocial intervention is available. Planned pregnancy was also another consistent predictor of remission, supporting previous evidence that pregnancy intentionality is associated with more favorable mental health outcomes during the perinatal period (37, 38).

Exposure to partner violence emerged as one of the strongest negative predictors of remission, particularly during the early postpartum period. This finding is consistent with extensive literature identifying intimate partner violence is an important risk factor for persistent perinatal depression and anxiety, with effects that may extend beyond pregnancy (39, 40). These findings highlight the importance of integrating mental health care with routine identification of intimate partner violence and accessible, confidential referral pathways to gender-based violence and other support services within antenatal and postnatal care.

Notably, educational attainment and employment status were not associated with remission at any time point. This contrasts with findings of studies in adult pregnant women in which education and employment status have been associated with postpartum remission of depressive symptoms (41). Among adolescent mothers, however, educational attainment and employment may be less informative indicators of socioeconomic resources because opportunities for education and employment are often constrained by age, pregnancy, caregiving responsibilities, and broader structural factors (32). Our findings therefore suggest that, within this adolescent population, relational and contextual factors including - age, marital status, pregnancy intentionality, living arrangements, and exposure to partner violence may play a more closely related to symptom remission than conventional individual socioeconomic indicators.

### Strengths

This study analyzed data from a large cohort of adolescent women attending antenatal clinics with a very low attrition rate of 3.4%, thereby minimizing the risk of bias due to loss to follow-up. To our knowledge, this study provides among the first longitudinal estimates of persistence and remission of depressive and generalized anxiety symptoms, as well as their associated factors, among adolescent mothers participating in a non-pharmacological intervention. These findings contribute to the limited evidence on the course of perinatal mental health symptoms among adolescent mothers in low-resource settings.

### Limitations

Several limitations should be acknowledged. Depressive and anxiety symptoms were assessed using screening instruments rather than structured diagnostic interviews, which may have resulted in misclassification. Nevertheless, adolescents with elevated symptoms in the absence of a formal psychiatric diagnosis represent a clinically important group warranting attention. Because adolescents were asked to report symptoms experienced over the preceding two weeks, recall bias may have also influenced prevalence estimates. In addition, the study was conducted in urban ANC health facilities in Mwanza, Tanzania, which may limit the generalizability of findings to rural populations and other health care settings. Repeated use of the same screening tools (EPDS and GAD-7) across assessment points may also have introduced measurement-related effects, including potential response and recall effects.

The analysis of factors associated with remission were univariable, and therefore represent unadjusted associations; they should not be interpreted as independent predictors of remission. In addition, convenience sampling from selected antenatal clinics may have introduced selection or healthy-attender bias and limits the generalizability of the findings to adolescent pregnant women who do not access antenatal care or who receive care in other settings. Finally, because the study was embedded within a psychosocial intervention, the observed remission rates may have been influenced by the intervention; and should not be interpreted as estimates of spontaneous or untreated remission. Future studies conducted under routine-care conditions, ideally with appropriate comparison groups, are needed to better characterize the natural course of perinatal depressive and anxiety symptoms among adolescent mothers.

## Conclusion

Overall, this study highlights the dynamic nature of perinatal depressive and anxiety symptoms among adolescent mothers participating in a non-pharmacological psychosocial intervention and underscores the importance of sustained psychosocial support across pregnancy and the postpartum period. Integrating routine mental health screening with accessible psychological support into standard antenatal and postnatal care has the potential to substantially reduce the burden of perinatal mental symptoms among adolescent mothers in low-resource settings. Early identification and targeted, context-sensitive interventions are particularly important for adolescents with persistent symptoms and those experiencing ongoing psychosocial stressors. Strengthening continuity of mental health care across pregnancy and the postpartum period may help reduce symptom chronicity and support longer-term maternal and child well-being.

## Data Availability

The raw data supporting the conclusions of this article will be made available by the authors, without undue reservation.
